# Supplementary material for: Western diet increases COVID-19 disease severity in the Syrian hamster
Source: bioRxiv. 2021 Jun 17:2021.06.17.448814. Preprint. [Version 1] doi: 10.1101/2021.06.17.448814 (PMC8219093; doi:10.1101/2021.06.17.448814)
Supplement: 1 [file NIHPP2021.06.17.448814V1-supplement-1.pdf]

bioRxiv preprint doi: https://doi.org/10.1101/2021.06.17.448814; this version posted June 17, 2021. The copyright holder for this preprint (which was not certified by peer review) is the author/funder. This article is a US Government work. It is not subject to copyright under 17 USC 105 and is also made available for use under a CC0 license.

Supplemental Material

Western diet increases COVID-19 disease severity in the Syrian hamster

Julia R. Port[1]*, Danielle R. Adney[1]*, Benjamin Schwarz[2], Jonathan E. Schulz[1], Daniel E. Sturdevant[3], Brian J. Smith[4], Victoria A. Avanzato[1], Myndi G. Holbrook[1], Jyothi N. Purushotham[1], Kaitlin A. Stromberg[2], Ian Leighton[2], Catharine M. Bosio[2], Carl Shaia[4], Vincent J. Munster[1]#

1. Laboratory of Virology, National Institute of Allergy and Infectious Diseases, National Institutes of Health, Hamilton, MT, USA
2. Laboratory of Bacteriology, National Institute of Allergy and Infectious Diseases, National Institutes of Health, Hamilton, MT, USA
3. Genomics Unit, Research Technologies Branch, National Institute of Allergy and Infectious Diseases, National Institutes of Health, Hamilton, MT, USA
4. Rocky Mountain Veterinary Branch, Division of Intramural Research, National Institutes of Health, Hamilton, MT, USA

*These authors contributed equally

#Corresponding author: Vincent Munster, email: vincent.munster@nih.gov

bioRxiv preprint doi: https://doi.org/10.1101/2021.06.17.448814; this version posted June 17, 2021. The copyright holder for this preprint (which was not certified by peer review) is the author/funder. This article is a US Government work. It is not subject to copyright under 17 USC 105 and is also made available for use under a CC0 license.

Supplemental Table 1: Liver marker profile in serum of regular diet (RD) and high-fat high-sugar diet (HFHS) after 16 weeks. Quantitative determination of total cholesterol (CHOL), high-density lipoprotein cholesterol (HDL), triglycerides (TRIG), alanine aminotransferase (ALT), aspartate aminotransferase (AST), and glucose (GLU) in heparinized whole blood. From the CHOL, HDL and TRIG determinations, low-density lipoprotein cholesterol (LDL), very low-density lipoprotein cholesterol (VLDL), non-HDL cholesterol, and a total cholesterol/high-density lipoprotein cholesterol ratio (TC/H) was calculated. ~~ + could not be calculated, LIP = not detectable due to lipid interference.

Animal ID	Chol mg/dl	HDL mg/dl	Trig mg/dl	ALT U/L	AST U/L	GLU mg/dl	nHDLc mg/dl	TC/H	LDL mg/dl	VLDL mg/dl	LIP
HFHS.1	360	LIP	~~~	124	78	120	~~~	~~~	LIP	LIP	3
HFHS.2	>520	HEM	HEM	224	HEM	LIP	~~~	~~~	~~~	~~~	3
HFHS.3	~~~	HEM	~~~	140	HEM	LIP	~~~	~~~	~~~	~~~	3
HFHS.4	>520	HEM	HEM	190	HEM	LIP	~~~	~~~	~~~	~~~	3
HFHS.5	380	LIP	>500	117	82	114	~~~	~~~	LIP	LIP	3
HFHS.6	263	LIP	>500	152	86	110	~~~	~~~	LIP	LIP	3
HFHS.7	384	~~~	~~~	188	147	LIP	~~~	~~~	~~~	~~~	3
HFHS.8	331	LIP	>500	114	84	59	~~~	~~~	LIP	LIP	2
Regular.1	83	49	212	62	76	113	34c	1.7c	0	42c	1
Regular.2	52	31	168	100	152	151	21c	1.7c	0	34c	0
Regular.3	98	68	248	67	79	72	30c	1.4c	0	50c	0
Regular.4	111	92	252	87	87	79	19c	1.2c	~~~	50c	1
Regular.5	74	43	184	73	96	98	31c	1.7c	0	37c	1
Regular.6	59	32	201	87	113	119	27c	1.8c	0	40c	1
Regular.7	43	23	204	148	127	101	20c	1.8c	~~~	41c	0
Regular.8	71	42	198	80	88	85	29c	1.7c	0	40c	0
Regular.9	64	46	232	142	157	88	18c	1.4c	~~~	46c	0
Regular.10	49	28	251	123	166	99	21c	1.7c	~~~	50c	0

bioRxiv preprint doi: https://doi.org/10.1101/2021.06.17.448814; this version posted June 17, 2021. The copyright holder for this preprint (which was not certified by peer review) is the author/funder. This article is a US Government work. It is not subject to copyright under 17 USC 105 and is also made available for use under a CC0 license.

Supplemental Table 2: Up-and down-regulated pathways in livers pre-challenge organized by disease and function.

Diseases or Functions Annotation	p-value	Predicted Activation State	Activation z-score	# Molecules
Development of genitourinary system	2.07E-11	Increased	2.114	160
Internalization of cells	1.99E-11	Increased	5.333	63
Abnormal bone density	1.99E-11	Decreased	-2.297	50
Phagocytosis of blood cells	1.89E-11	Increased	4.815	52
Adhesion of lymphocytes	1.54E-11	Increased	3.747	34
Interaction of T lymphocytes	1.36E-11	Increased	3.906	39
Adhesion of lymphatic system cells	1.33E-11	Increased	3.839	35
Cell movement of macrophages	1.32E-11	Increased	4.236	65
Adhesion of tumor cell lines	9.66E-12	Increased	2.42	68
Pancreatobiliary tumor	9.07E-12	Increased	2.146	402
Size of body	9.03E-12	Increased	2.966	126
Cell cycle progression	8.08E-12	Increased	2.341	171
Binding of T lymphocytes	7.98E-12	Increased	3.521	37
Interaction of lymphocytes	5.70E-12	Increased	3.973	45
Pancreatic lesion	5.57E-12	Increased	2.114	358
Migration of neutrophils	5.56E-12	Increased	3.941	38
Response of myeloid leukocytes	5.43E-12	Increased	2.565	34
Chemotaxis of neutrophils	5.10E-12	Increased	2.328	42
Migration of granulocytes	4.82E-12	Increased	3.2	43
Cell-cell contact	4.15E-12	Increased	2.864	136
Development of head	3.98E-12	Increased	3.325	164
Quantity of metal ion	3.75E-12	Increased	2.912	85
Aggregation of blood platelets	3.23E-12	Increased	3.084	49
Immune response of antigen presenting cells	3.23E-12	Increased	3.968	49
Binding of lymphatic system cells	2.58E-12	Increased	3.801	45
Transmigration of leukocytes	2.57E-12	Increased	2.603	42
Binding of lymphocytes	2.47E-12	Increased	3.619	43
Transport of molecule	2.41E-12	Increased	2.741	246
Malignant connective or soft tissue neoplasm	2.36E-12	Increased	2.079	203
Response of antigen presenting cells	1.93E-12	Increased	4.049	52
Recruitment of macrophages	1.92E-12	Increased	2.61	33

bioRxiv preprint doi: https://doi.org/10.1101/2021.06.17.448814; this version posted June 17, 2021. The copyright holder for this preprint (which was not certified by peer review) is the author/funder. This article is a US Government work. It is not subject to copyright under 17 USC 105 and is also made available for use under a CC0 license.

Engulfment of cells	1.87E-12	Increased	4.959	99
Phagocytosis	1.52E-12	Increased	5.163	81
Homing of neutrophils	1.47E-12	Increased	2.328	43
Phagocytosis of cells	1.45E-12	Increased	5.586	75
Binding of endothelial cells	1.13E-12	Increased	2.496	49
Transmigration of cells	1.13E-12	Increased	2.844	49
Cell movement of cancer cells	8.81E-13	Increased	2.68	41
Interaction of endothelial cells	8.80E-13	Increased	2.389	50
Cellular infiltration by myeloid cells	8.74E-13	Increased	2.023	72
Inflammation of respiratory system component	7.19E-13	Increased	2.017	105
Binding of lymphoid cells	7.09E-13	Increased	3.713	44
Activation of antigen presenting cells	6.15E-13	Increased	3.438	70
Homeostasis of blood cells	5.80E-13	Increased	3.757	111
Activation of myeloid cells	4.67E-13	Increased	3.734	74
Degranulation of phagocytes	4.12E-13	Increased	3.533	90
Activation of phagocytes	3.71E-13	Increased	3.826	79
Engulfment of myeloid cells	3.71E-13	Increased	4.655	47
Degranulation of myeloid cells	3.58E-13	Increased	3.647	91
Synthesis of reactive oxygen species	2.88E-13	Increased	4.241	99
Homeostasis of leukocytes	2.75E-13	Increased	3.757	110
Malignant neoplasm of retroperitoneum	2.37E-13	Increased	2.021	419
Quantity of Ca2+	2.14E-13	Increased	2.613	82
Engulfment of leukocytes	2.00E-13	Increased	4.233	51
Recruitment of myeloid cells	1.80E-13	Increased	4.402	64
T cell development	1.58E-13	Increased	3.822	104
Degranulation of leukocytes	1.56E-13	Increased	3.4	95
Upper gastrointestinal tract tumor	1.49E-13	Increased	2.236	581
Invasion of cells	1.48E-13	Increased	4.617	184
Engulfment of phagocytes	1.43E-13	Increased	4.266	49
Cell movement of tumor cell lines	1.37E-13	Increased	4.127	185
Metabolism of reactive oxygen species	1.34E-13	Increased	4.381	104
Growth of connective tissue	1.31E-13	Increased	2.459	123
Amyloidosis	1.30E-13	Decreased	-2.433	116
Activation of mononuclear leukocytes	1.19E-13	Increased	3.257	95

bioRxiv preprint doi: https://doi.org/10.1101/2021.06.17.448814; this version posted June 17, 2021. The copyright holder for this preprint (which was not certified by peer review) is the author/funder. This article is a US Government work. It is not subject to copyright under 17 USC 105 and is also made available for use under a CC0 license.

Upper gastrointestinal tract cancer	1.13E-13	Increased	2	580
Lymphopoiesis	9.10E-14	Increased	3.693	123
Adhesion of mononuclear leukocytes	7.27E-14	Increased	3.616	42
Quantity of immunoglobulin	6.65E-14	Increased	3.038	63
Production of antibody	6.01E-14	Increased	3.204	66
Cell viability	5.81E-14	Increased	4.969	235
Phagocytosis of phagocytes	4.62E-14	Increased	4.14	46
Phagocytosis of leukocytes	4.60E-14	Increased	4.229	47
Recruitment of antigen presenting cells	4.55E-14	Increased	3.038	38
Phagocytosis of myeloid cells	3.83E-14	Increased	4.396	46
Activation of lymphocytes	3.64E-14	Increased	3.171	93
Cell movement of T lymphocytes	3.42E-14	Increased	3.59	63
Recruitment of phagocytes	3.01E-14	Increased	4.827	62
Cell survival	1.69E-14	Increased	4.795	247
Activation of lymphoid cells	1.50E-14	Increased	3.25	94
Migration of myeloid cells	1.43E-14	Increased	3.972	54
Production of protein	1.43E-14	Increased	3.73	70
Quantity of B lymphocytes	1.29E-14	Increased	2.549	81
Activation of lymphatic system cells	1.09E-14	Increased	3.088	95
Metastasis	1.03E-14	Increased	3.523	181
Interaction of mononuclear leukocytes	9.60E-15	Increased	3.685	55
Growth of epithelial tissue	7.66E-15	Increased	2.312	135
Quantity of T lymphocytes	7.33E-15	Increased	4.155	111
T cell homeostasis	7.05E-15	Increased	3.667	109
Hematopoiesis of mononuclear leukocytes	6.16E-15	Increased	3.759	132
Migration of antigen presenting cells	4.15E-15	Increased	3.735	51
Immediate hypersensitivity	4.06E-15	Increased	2.362	77
Response of myeloid cells	3.82E-15	Increased	4.283	60
Immune response of myeloid cells	2.85E-15	Increased	4.069	56
Non-colon gastrointestinal cancer	2.59E-15	Increased	2	612
Aggregation of cells	2.51E-15	Increased	3.773	77
Extraadrenal retroperitoneal tumor	2.51E-15	Increased	2.58	463
Chemotaxis of myeloid cells	2.36E-15	Increased	3.493	70
Leukopoiesis	2.22E-15	Increased	4.158	149

bioRxiv preprint doi: https://doi.org/10.1101/2021.06.17.448814; this version posted June 17, 2021. The copyright holder for this preprint (which was not certified by peer review) is the author/funder. This article is a US Government work. It is not subject to copyright under 17 USC 105 and is also made available for use under a CC0 license.

Aggregation of blood cells	1.69E-15	Increased	3.56	62
Differentiation of mononuclear leukocytes	1.67E-15	Increased	3.796	134
Hereditary connective tissue disorder	1.43E-15	Decreased	-3.259	128
Binding of mononuclear leukocytes	1.33E-15	Increased	3.331	54
Advanced malignant tumor	8.44E-16	Increased	3.434	197
Connective tissue tumor	7.45E-16	Increased	2.495	222
Advanced stage tumor	5.25E-16	Increased	3.434	198
Chemotaxis of phagocytes	4.47E-16	Increased	3.987	73
Recruitment of blood cells	4.05E-16	Increased	4.395	79
Connective or soft tissue tumor	3.53E-16	Increased	2.525	249
Degranulation of cells	3.26E-16	Increased	3.573	115
Recruitment of leukocytes	2.92E-16	Increased	4.208	78
Chemotaxis of leukocytes	2.63E-16	Increased	4.146	84
Immune response of phagocytes	2.22E-16	Increased	4.017	62
Growth of tumor	2.05E-16	Increased	4.005	179
Degranulation	1.09E-16	Increased	3.507	117
Inflammation of joint	1.07E-16	Increased	3.397	178
Response of phagocytes	9.69E-17	Increased	4.268	66
Chemotaxis of blood cells	9.45E-17	Increased	4.147	85
Recruitment of cells	8.37E-17	Increased	4.692	85
T cell migration	7.55E-17	Increased	4.271	73
Immune response of leukocytes	7.50E-17	Increased	4.727	80
Hypersensitive reaction	6.65E-17	Increased	3.831	97
Vasculogenesis	5.92E-17	Increased	3.35	158
Angiogenesis	5.56E-17	Increased	4.04	184
Development of vasculature	4.93E-17	Increased	3.955	198
Binding of tumor cell lines	4.04E-17	Increased	2.518	93
Homing of leukocytes	3.51E-17	Increased	4.4	89
Cell movement of antigen presenting cells	2.39E-17	Increased	4.024	91
Binding of myeloid cells	2.30E-17	Increased	3.053	60
Cellular homeostasis	2.19E-17	Increased	4.676	269
Experimental autoimmune encephalomyelitis	1.82E-17	Increased	3.58	86
Homing of blood cells	1.80E-17	Increased	4.405	90
Interaction of tumor cell lines	1.57E-17	Increased	2.283	96
Microtubule dynamics	6.51E-18	Increased	3.927	216
Cell movement of granulocytes	5.63E-18	Increased	3.593	93

bioRxiv preprint doi: https://doi.org/10.1101/2021.06.17.448814; this version posted June 17, 2021. The copyright holder for this preprint (which was not certified by peer review) is the author/funder. This article is a US Government work. It is not subject to copyright under 17 USC 105 and is also made available for use under a CC0 license.

Cell movement of lymphatic system cells	2.90E-18	Increased	4.135	101
Organization of cytoplasm	2.90E-18	Increased	4.437	262
Cell movement of neutrophils	2.55E-18	Increased	3.635	83
Encephalitis	2.39E-18	Increased	2.541	95
Immune response of cells	1.57E-18	Increased	4.854	130
Cell movement of lymphocytes	1.14E-18	Increased	4.249	100
Allergy	5.20E-19	Increased	3.26	98
Rheumatic Disease	4.02E-19	Increased	3.322	223
Organismal death	3.96E-19	Decreased	-4.3	362
Morbidity or mortality	2.66E-19	Decreased	-4.307	366
Cell proliferation of T lymphocytes	2.10E-19	Increased	2.415	127
Development of body trunk	1.36E-19	Increased	3.651	215
Binding of professional phagocytic cells	1.35E-19	Increased	2.925	61
Inflammation of central nervous system	1.33E-19	Increased	2.333	102
Invasive tumor	1.09E-19	Increased	3.538	224
Migration of lymphatic system cells	8.48E-20	Increased	4.544	95
Lymphocyte migration	5.97E-20	Increased	4.62	94
Organization of cytoskeleton	5.36E-20	Increased	4.437	249
Chemotaxis	4.82E-20	Increased	4.815	123
Migration of mononuclear leukocytes	3.71E-20	Increased	4.931	99
Proliferation of lymphocytes	3.65E-20	Increased	3.292	150
Proliferation of immune cells	3.58E-20	Increased	3.303	158
Proliferation of lymphatic system cells	3.30E-20	Increased	3.653	159
Homing of cells	2.85E-20	Increased	4.933	128
Interaction of phagocytes	2.61E-20	Increased	3.318	64
Quantity of lymphatic system cells	1.12E-20	Increased	4.27	161
Proliferation of blood cells	8.94E-21	Increased	2.84	172
Migration of phagocytes	7.21E-21	Increased	5.059	82
Proliferation of mononuclear leukocytes	3.68E-21	Increased	3.384	154
Quantity of lymphocytes	1.41E-21	Increased	4.164	155
Cell movement of mononuclear leukocytes	7.82E-22	Increased	4.793	119
Quantity of lymphoid cells	7.71E-22	Increased	4.267	156
Cell movement of myeloid cells	9.41E-23	Increased	5.148	136
Atherosclerosis	5.83E-23	Increased	2.673	111

bioRxiv preprint doi: https://doi.org/10.1101/2021.06.17.448814; this version posted June 17, 2021. The copyright holder for this preprint (which was not certified by peer review) is the author/funder. This article is a US Government work. It is not subject to copyright under 17 USC 105 and is also made available for use under a CC0 license.

Arteriosclerosis	5.55E-23	Increased	2.673	112
Cancer of cells	5.08E-23	Increased	2.324	684
Occlusion of artery	8.44E-24	Increased	2.291	124
Activation of leukocytes	7.41E-24	Increased	3.906	152
Quantity of mononuclear leukocytes	3.21E-24	Increased	4.23	166
Development of digestive organ tumor	2.85E-24	Increased	2.223	805
Occlusion of blood vessel	1.78E-24	Increased	2.439	127
Cell movement of phagocytes	2.32E-25	Increased	5.16	143
Vaso-occlusion	1.80E-25	Increased	2.762	130
Inflammatory response	5.12E-26	Increased	4.721	179
Neoplasia of cells	4.32E-26	Increased	3.343	764
Nervous system neoplasm	1.09E-26	Increased	2.631	863
Binding of leukocytes	6.17E-27	Increased	4.377	107
Adhesion of immune cells	3.52E-27	Increased	4.659	102
Activation of blood cells	2.67E-27	Increased	4.18	171
Binding of blood cells	1.99E-28	Increased	4.151	117
Activation of cells	1.77E-28	Increased	4.235	212
Adhesion of blood cells	1.50E-29	Increased	4.576	110
Cell movement of leukocytes	2.69E-31	Increased	5.68	193
Leukocyte migration	2.22E-31	Increased	6.163	217
Quantity of cells	2.80E-32	Increased	4.371	346
Migration of cells	2.28E-33	Increased	5.915	387
Quantity of leukocytes	8.59E-34	Increased	3.627	215
Quantity of blood cells	1.14E-34	Increased	3.879	234
Cell movement	1.12E-34	Increased	6.237	422
Digestive organ tumor	2.14E-45	Increased	2.245	1243
Intraabdominal organ tumor	1.16E-50	Increased	2.485	1285
Cancer	3.98E-60	Increased	3.984	1378
Solid tumor	1.91E-61	Increased	2.377	1380
Malignant solid tumor	6.41E-62	Increased	2.227	1377
Non-melanoma solid tumor	2.74E-65	Increased	2.163	1366

bioRxiv preprint doi: https://doi.org/10.1101/2021.06.17.448814; this version posted June 17, 2021. The copyright holder for this preprint (which was not certified by peer review) is the author/funder. This article is a US Government work. It is not subject to copyright under 17 USC 105 and is also made available for use under a CC0 license.

A

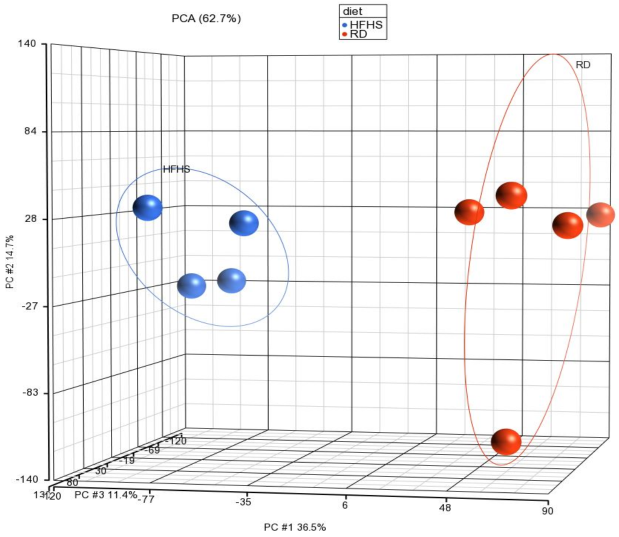

Supplemental Figure 1: A. RNA was isolated for gene expression analyses from liver tissue at 16 weeks and principal component analysis performed. Colors refer to legend on top. Abbreviations: RD = regular diet, HFHS = high-fat high-sugar, PC = principal component.

A B

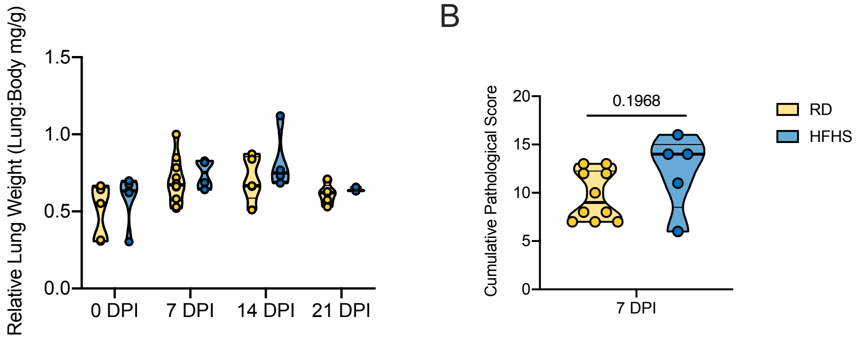

Supplemental Figure 2: Male Syrian hamsters were fed either a regular or high-fat high-sugar *diet ad libitum* for 16 weeks, then challenged with $8x10^4$ $TCID_{50}$ SARS-CoV-2. Animals were euthanized pre-challenge (0 DPI), at 7, 14 and 21 DPI. **A.** Lung weights. Truncated violin plots depicting median, quartiles, and individuals. **B.** Cumulative pathology score of lung tissues collected at 7 DPI. Truncated violin plots depicting median, quartiles and individuals, N = 10 (RD) / 4 (HFHS), Mann-Whitney test. Abbreviations: RD = regular diet, HFHS = high-fat high-sugar. p-values are indicated were appropriate.

bioRxiv preprint doi: https://doi.org/10.1101/2021.06.17.448814; this version posted June 17, 2021. The copyright holder for this preprint (which was not certified by peer review) is the author/funder. This article is a US Government work. It is not subject to copyright under 17 USC 105 and is also made available for use under a CC0 license.

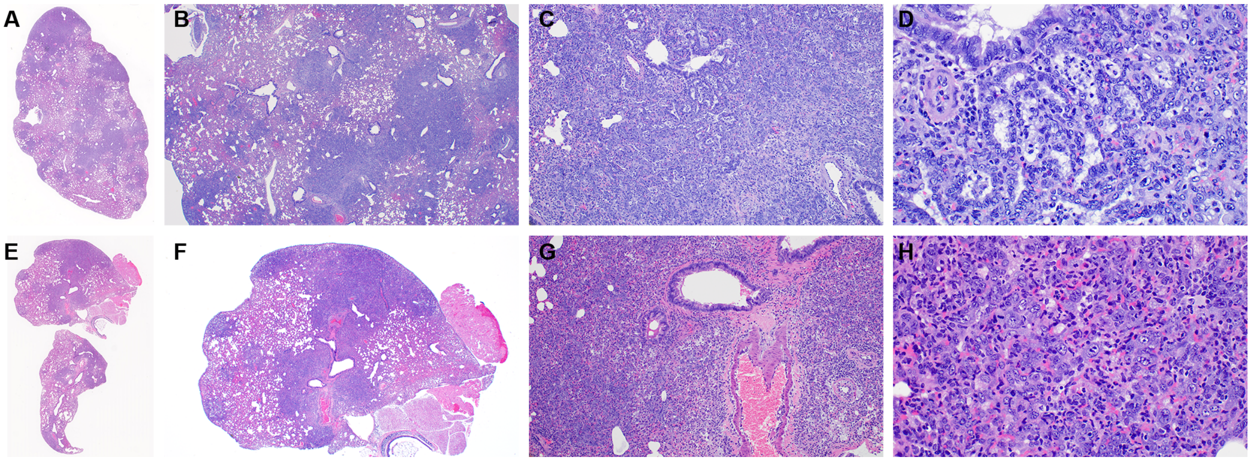

Supplemental Figure 3: Male Syrian hamsters were fed either a regular or high-fat high-sugar *diet ad libitum* for 16 weeks, then challenged with $8x10^4$ $TCID_{50}$ SARS-CoV-2. Animals were euthanized at day 8 and 9 due to increased weight loss. **A, E.** Dark, discreet foci identify areas of pneumonia; lighter areas indicate hemorrhage, edema, inflammation. HE, 1.4x. **B, F.** Although approximately 100% of the lobe is affected, only 50% contains discreet foci of interstitial pneumonia, HE, 20x. **C, D.** Examples of organized type II pneumocyte hyperplasia giving a honeycomb appearance. HE, 100x, 400x. **G, H.** Less well organized foci with more congestion, edema, and inflammation. HE, 100x, 400x. Of note, both appearances overlap and can be present in the same animal.

bioRxiv preprint doi: https://doi.org/10.1101/2021.06.17.448814; this version posted June 17, 2021. The copyright holder for this preprint (which was not certified by peer review) is the author/funder. This article is a US Government work. It is not subject to copyright under 17 USC 105 and is also made available for use under a CC0 license.

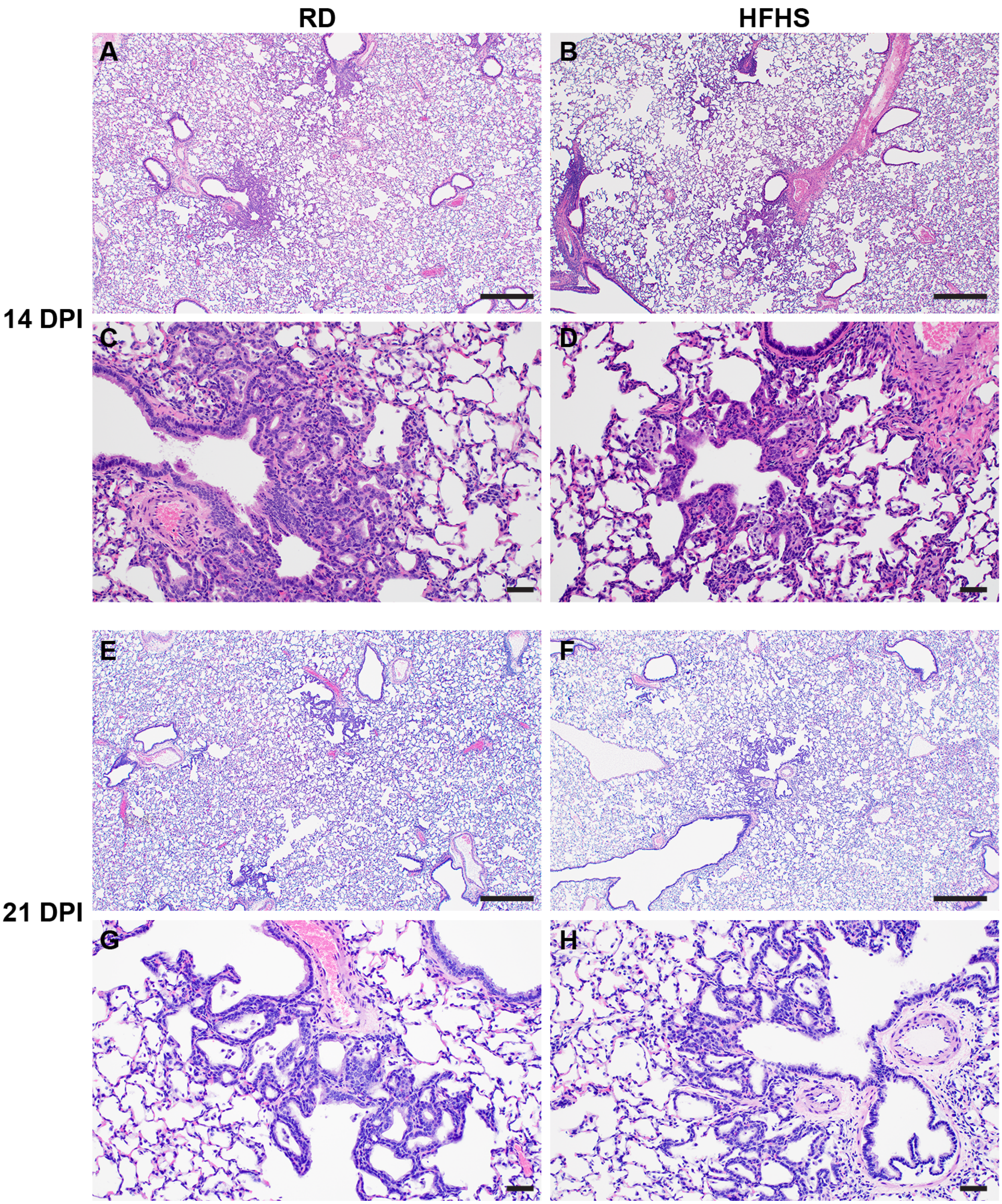

Supplemental Figure 4: Male Syrian hamsters were fed either a regular or high-fat high-sugar *diet ad libitum* for 16 weeks, then challenged with $8x10^4$ $TCID_{50}$ SARS-CoV-2. Lung tissues were collected 14 and 21 days post inoculation. **A, B**. 14 DPI, Lesions located at terminal bronchioles.

bioRxiv preprint doi: https://doi.org/10.1101/2021.06.17.448814; this version posted June 17, 2021. The copyright holder for this preprint (which was not certified by peer review) is the author/funder. This article is a US Government work. It is not subject to copyright under 17 USC 105 and is also made available for use under a CC0 license.

HE, 40x. **C, D**. 14 DPI, Thickened septa, alveolar bronchiolization and minimal inflammation. HE, 400x. **E, F**. 21 DPI, Lesions appear indistinguishable. HE, 40x. **G, H**. 21 DPI, Thickened septa and alveolar bronchiolization remain. HE, 400x. Abbreviations: Reg = regular diet, HFHS = high-fat high-sugar, DPI = days post inoculation.

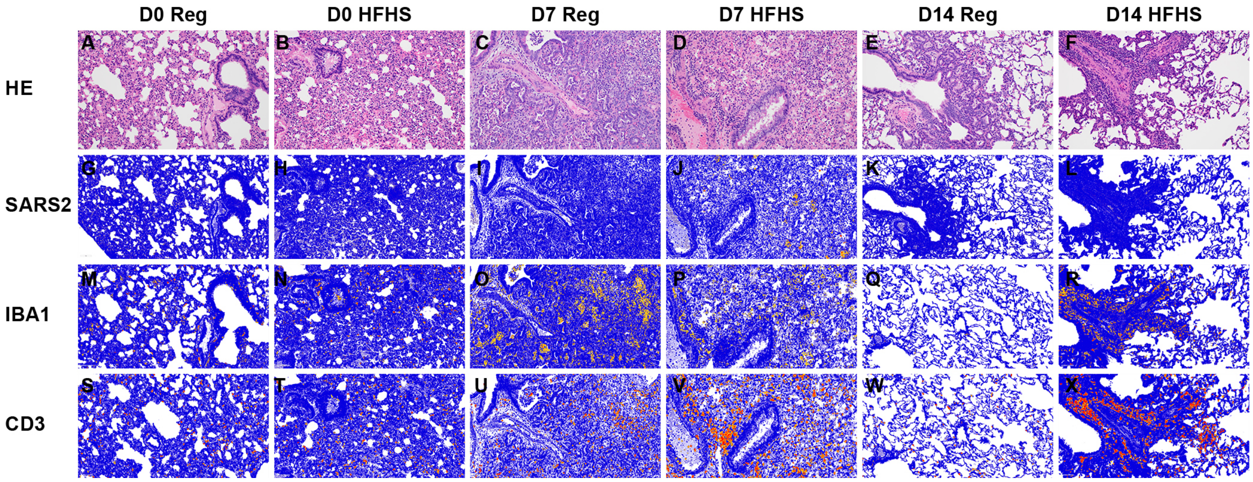

Supplemental Figure 5: Male Syrian hamsters were fed either a regular or high-fat high-sugar *diet ad libitum* for 16 weeks, then challenged with 8x10^4 $TCID_{50}$ SARS-CoV-2. Animals were euthanized pre-challenge (0 DPI), 7 and 14 days post inoculation. Serial images of lungs. **A-F**. Pre-challenge lungs appear normal, 7 DPI lungs are pneumonic, and 14 DPI lungs appear to be resolving. HE, 200x. **G-L.** Positive pixel image of IHC staining against N protein of SARS-CoV-2. Note the positive pixels at 7 DPI in the HFHS image, 200x. **M-P.** Positive pixel image of IHC staining against IBA1. Note the increase in positive pixels at 7 and 14 DPI for both the RD and HFHS samples, 200x. **Q-X.** Positive pixel image of IHC staining against CD3, Note the increase in positive pixels at 7 and 14 DPI for both the RD and HFHS samples, 200x. Positive pixel = orange. Abbreviations: Reg = regular diet, HFHS = high-fat high-sugar, DPI = days post inoculation.
